# Reader Comment Regarding: Pediatric Dermatology in Canada: A Broad Review of Population Needs, Workforce, and Training With Proposed Solutions

**DOI:** 10.1177/12034754241255341

**Published:** 2024-05-20

**Authors:** Evert Tuyp

**Affiliations:** 1 Department of Dermatology and Skin Science, The University of British Columbia, Vancouver, BC, Canada

Dear Editor,

Pope et al present a welcome discussion on pediatric dermatology in Canada.^1^ Their article states that “across Canada, the recommended ratio of dermatologists to population is 1:62,650, but the ratio is only met in Quebec (In Depth Review of Dermatology Training and Practice, September 2019).” There are a few issues with this statement that require clarification and discussion. First, they did not include this citation in their references and so I can only guess that they were referring to “An in-depth review of Dermatology training and practice in Canada” published by the Royal College of Physicians and Surgeons of Canada Committee for Specialties and submitted by Mydlarski, Chair of the Specialty Committee in Dermatology, September 2019.^2^ The sourcing of this reference is problematic but fortunately a 2-part series was published by members of this group in this same *Journal of Cutaneous Medicine and Surgery (JCMS)*.^3,4^ These articles reviewed a lot of the same topics that Pope’s group discuss. The first article brought up the 1:62,650 ratio also, but more important, it referenced this ratio back to its origins in the Royal College of Physician and Surgeon’s National Specialty Physician Review (NSPR) of 1988.^5^ That was a long time ago—before the explosion of cosmetic dermatology (fillers, Botox, lasers, etc). Consequently, the validity of this ratio is very suspicious, a point which was made following the publication of the initial article by Mydlarski in the *JCMS* in 2019.^6,7^ Furthermore, the very long wait times to see a dermatologist in Quebec suggest a lack of patient access to dermatologic care and an actual dermatologist shortage as opposed to adequate supply of dermatologists as suggested by data that Quebec meets the recommended NSPR ratio.^8^

The arguments regarding this ratio and ancillary issues have been explored already and it is important to attach the relevant references to Pope et al’s article for the benefit of those who have an interest in the dermatology workforce in Canada.
